# Assessing the Utility of AI Versus Human-Created MCQs in Pediatric Medical Education

**DOI:** 10.1177/23821205261427885

**Published:** 2026-03-03

**Authors:** James Knight, Richard G. McGee, Bunmi S. Malau-Aduli, for the ACHIEVE network

**Affiliations:** 1 625133 Faculty of Medicine and Health, School of Rural Health, The University of New England, Armidale, NSW, Australia; 2 64834 School of Medicine and Public Health, College of Health, Medicine and Wellbeing, University of Newcastle, Newcastle, NSW, Australia; 3 John Hunter Children's Hospital, New Lambton, NSW, Australia; 4 Hunter Medical Research Institute, New Lambton, NSW, Australia

**Keywords:** artificial intelligence, multiple-choice questions, item analysis, pediatrics, medical education, assessment validity

## Abstract

**Background:**

Multiple-choice questions (MCQs) remain central to assessment in medical education, but their development is resource intensive. Generative artificial intelligence (AI) offers a potential solution by automating MCQ creation. However, little is known about the psychometric quality of AI-generated MCQs compared with human-authored items, particularly in pediatric education.

**Objective:**

This study aimed to directly compare the quality of AI- and human-generated MCQs in pediatrics using item analysis grounded in classical test theory.

**Methods:**

A formative exam comprising both AI (Microsoft Copilot) and human-generated pediatric MCQs was administered to 4th-year medical students. Item analysis was performed to calculate difficulty indices, discrimination indices, item-total correlations, and distractor functioning. Reliability was assessed using KR-20. Descriptive and inferential statistics, including paired *t*-tests, compared performance between AI and human items.

**Results:**

Human-authored questions outperformed AI-generated questions across all quality indicators. AI questions showed lower discrimination (mean 0.19 vs. 0.29) and a higher proportion outside the acceptable difficulty range (56% vs. 32%). Distractor analysis also favored human questions, with fewer nonfunctioning distractors and more items containing fully functional distractors. While some AI items met ideal psychometric thresholds, overall consistency was lower.

**Conclusion:**

Generative AI in its current form cannot yet match human expertise in producing consistently high-quality MCQs for pediatrics. However, AI shows potential as a supplementary tool, particularly within hybrid human–AI workflows that combine efficiency with expert oversight. These findings highlight both the opportunities and limitations of AI in medical education assessment and underscore the importance of balancing reliability, validity, acceptability, and cost-effectiveness when integrating AI into assessment design.

## Introduction

Effective assessment strategies are crucial in medical education to ensure that future healthcare professionals possess the necessary knowledge, skills, and competencies to practice safely and effectively.^1,2^ Multiple choice questions (MCQs) are a principal component of educational assessments globally, due to their objectivity, reliability, feasibility, ease of administration, ability to test higher order knowledge and to efficiently assess a broad range of content knowledge among large student cohorts.^3,4^ MCQ exams are the most common assessment method in medical education and remain a valuable method for assessment of knowledge-based curriculum aspects and the lower levels of Miller's pyramid.^5^ The role of MCQs within the medical education setting has also expanded beyond an assessment tool, with the development of MCQs by students having been shown to enhance learning.^6^ Repeated testing with MCQs as a way to facilitate a spaced-repetition revision method which promotes recurrent recall of information has also been demonstrated to improve student retention of content and improved examination results when compared to passive study methods.^7–10^ MCQs, when meticulously designed, offer reliable measurement of knowledge recall, comprehension, application, and clinical reasoning abilities.^11^ However, the rigorous construction of high-quality MCQs is resource-intensive, requiring considerable faculty expertise, training, and time.^12^

Recent advancements in artificial intelligence (AI), particularly large language models such as ChatGPT, GPT-4, and other generative AI platforms, have sparked significant interest and introduced novel possibilities in educational assessment by automating the creation of MCQs.^13–15^ Generative AI has been shown to be able to generate MCQs up to 10 times faster than human assessors.^14^ This represents a significant opportunity for examiners, as MCQ questions are often time consuming to generate manually, particularly for inexperienced item writers, for whom a single high-quality question can take over 90 min to create.^16^ AI-generated questions offer potential advantages including rapid question production, scalability, and consistent adherence to predefined structural guidelines.^17–19^ Despite these promising attributes, concerns persist regarding the accuracy, validity, and discriminative ability of AI-generated MCQs, especially when assessing complex clinical domains, where nuanced clinical reasoning and context-specific judgment are critical.^20^

In the context of pediatric medical education, assessment quality is particularly critical due to the nuanced understanding required for diagnosis and management of pediatric conditions, the variability of clinical presentations, and the necessity for precision and sensitivity in clinical decision-making.^21,22^ Given these complexities, assessing whether AI-generated MCQs match the educational validity, reliability, and clinical relevance of those crafted by experienced human medical educators is paramount.

Preliminary studies evaluating the efficacy of AI-generated MCQs have yielded mixed findings.^14,23^ While some studies report that AI-generated items are comparable to human-authored questions in terms of item difficulty and discrimination,^24^ others highlight significant limitations such as inaccuracies in content representation, simplistic clinical scenarios, or inadequate distractors that fail to appropriately challenge examinees.^25–27^ Moreover, direct comparative analyses between AI-generated and human-authored questions in medical education, particularly in pediatrics, remain sparse.

To address this gap, the current study aimed to directly compare AI-generated and human-generated MCQs on pediatric medicine content through a formative assessment administered to medical students. This study employed rigorous item analysis methods, including classical test theory (CTT) parameters to objectively quantify and contrast the strengths and shortcomings of AI-generated MCQs against traditional human-authored MCQs.^1,28^ Specifically, this study aimed to answer the following research questions:

1. How do AI-generated MCQs perform relative to human-generated MCQs in terms of item difficulty, discrimination power, and distractor efficacy?
2. What are the distinctive strengths and shortcomings of AI-generated questions as compared to those developed by experienced pediatric educators?

## Methodology

This study employed a cross-sectional comparative design to assess the psychometric quality of AI-generated and human-authored pediatric MCQs which were developed in March 2024. The resulting 50-item examination was administered in October 2024, as a voluntary, formative online assessment to undergraduate medical students enrolled at the University of Newcastle and University of New England in Australia. Ethics approval (QA286) was obtained from the University of Newcastle's Research Ethics and Integrity Unit. All students participated voluntarily and signed a declaration to be included in the study.

### Development of the test items

Microsoft Copilot (a free online generative AI program based on GPT-4) was used to generate 120 MCQs based on common pediatrics content aimed at undergraduate medical students, 25 questions were included in the final exam.^29^ Copilot was selected due to being freely and widely accessible at the time of generation (March 2024) making the results more generalizable than a specialized trained AI program. The generative AI model was prompted using the following script:

“*can you write me 20 MCQs with answers on common paediatrics topics appropriate to the level of a 4^th^ year medical student*,” further prompting asked the model to generate additional questions on specific topic areas such as “*write me 10 more MCQs about infectious diseases in pediatrics.*” No reference materials were provided to the model, and no additional prompting was used. One hundred twenty questions were generated to ensure that there was sufficient breadth of content as the AI model generates a large number of repeated questions.

Included questions were selected by an examiner with a focus on choosing questions which contained content which was accurate and relevant to the exam and covered a broad range of topics to adequately test the students. Questions were not selected based on perceived quality, however, there was human influence in the selection of the AI questions which were presented to the students. No edits were made to the final questions apart from correcting spelling and grammar and reordering the answer options into alphabetical order.

The final AI-generated MCQs were then reviewed by a consultant pediatrician with experience in assessment item generation who acted as a content expert to ensure questions were appropriate and the suggested answers were correct. Questions were generated in this way to leverage the AI tool's ability to generate large amounts of content in a short period of time as well as to create questions which reflected the capability of the AI with minimal human input. Twenty-five^25^ “human-generated” MCQs were also written by a consultant pediatrician with experience writing formal MCQ assessment items for medical students, these questions did not undergo a formal peer review process. All questions were single best answer type MCQs with five answer options. The examined content was based on the learning objectives set out by the university curriculum of the students who sat the exam.

### Administration of the test

The human- and AI-generated questions were then randomized into a 50 question MCQ exam using a random number generator. The exam was made available online to all medical students enrolled at the University of Newcastle and University of New England in their 4th year of study who were preparing for formal end of year assessment in October 2024. Students were given a 1-min time limit to answer each question and were unable to backtrack or change their answers after the time had expired to limit the use of external resources to assist with answering the questions. One point was awarded for a correct answer with no negative marking.

### Psychometric measurements and statistical analysis

Item analysis was conducted for the assessment using CTT, calculating the difficulty index (DIF), discrimination index (DI), item-total correlation coefficients (RPB), distractor efficiency and internal consistency reliability.^30^ DIF was defined as the proportion of students answering an item correctly, and DI was assessed using the discrimination index, reflecting an item's ability to differentiate between high- and low-performing students.^11,31^ RPB were calculated to assess the relationship between individual item performance and total test score.^30^ Distractor efficiency was evaluated by identifying nonfunctioning distractors, defined as options selected by fewer than 5% of examinees.^11,31^ Internal consistency reliability was assessed using the Kuder–Richardson Formula 20 (KR-20), which is appropriate for dichotomously scored items.^30^ Quality thresholds for these metrics followed criteria reported by Malau-Aduli and Zimitat (2012): acceptable DIF values were defined by *P*-values from ≥30% to <70%; DI values of 0 to 0.19 indicated nondiscriminating items, whereas values from 0.2 to 1.0 indicated discriminating items.^23,32^ Nonfunctioning distractors were identified as options selected by fewer than 5% of examinees.^32^ Frequency distributions were developed to categorize items based on their number of functioning distractors (ranging from 0 to 4).

Independent-samples *t*-tests were performed using SPSS version 29 (IBM Corp., Armonk, NY, USA) to compare item-level psychometric properties of AI-generated and human-generated MCQs. Analyses were conducted for item difficulty, discrimination index, point-biserial correlation, and number of functioning distractors per item. Items were treated as independent observational units. Effect sizes were calculated using Cohen's d. Statistical significance was set at *P* < .05.

## Results

The online test was taken by a total of 159 4th-year medical students, with each question item attracting a minimum of 152 responses.

### Item discrimination

The mean discrimination index for the examination overall was 0.25. AI-generated MCQs demonstrated discrimination indices ranging from −0.19 to 0.55 (see example of negatively discriminating AI generated question below), with a mean ± SD of 0.19 ± 0.17, whereas human-generated MCQs ranged from 0.04 to 0.66, with a higher mean ± SD of 0.29 ± 0.14 (Table 1). Independent-samples *t*-testing indicated that human-authored items had significantly higher discrimination indices than AI-generated items (*t*(45.7) = 2.21, *P* = .03), with a moderate effect size (Cohen's d = 0.63). More than half of the AI-generated items (56%) failed to meet the acceptable discrimination threshold of 0.20, compared with 26% of human-generated items. Three AI-generated questions had negative discrimination indices. When comparing the 5 most discriminating items (top quintile) in each group, mean discrimination was comparable but was lower for AI-generated questions (0.44) than for human-generated questions (0.49). In contrast, the 5 least discriminating AI-generated items showed a markedly lower mean discrimination (−0.07) compared with their human-authored counterparts (0.11).[Table table5-23821205261427885]

**Table table5-23821205261427885:** 

An example of a negatively discriminating AI generated question (DI −0.07)
A newborn infant presents with lethargy, poor feeding, and jaundice within the first few days of life. Laboratory investigations reveal elevated serum bilirubin levels and indirect hyperbilirubinemia. There is evidence of hemolysis on peripheral blood smear. Which of the following conditions is most likely causing the infant's presentation? ABO incompatibility Biliary atresia G6PD deficiency **Rh hemolytic disease of the newborn** Sepsis

**Table 1.** Item Discrimination Indices for AI-Generated and Human-Generated Pediatric Multiple-Choice Questions Administered to Participants.

	Human-Generated	AI-Generated
Number of items in exam	25	25
Number of examinees	159	159
Mean discrimination index (SD)	0.29 (0.14)	0.19 (0.17)
Items with negative discrimination indices (%)	0	3 (12%)
Items with zero discrimination indices (%)	0	0
Items with low discrimination indices (%)	6 (24%)	11 (44%)
Total number of discriminating items (%)	19 (76%)	14 (56%)

*Note*. Item discrimination index reflects an item's ability to differentiate between higher- and lower-performing students. Discrimination indices ≥ 0.20 were considered acceptable.

### Item–total correlations (RPB)

Item–total point-biserial correlation coefficients (RPB) were examined as an additional indicator of item quality and alignment with overall test performance. AI-generated items demonstrated lower mean RPB values (mean ± SD: 0.17 ± 0.18) compared with human-generated items (0.23 ± 0.15) (Table 2). Independent-samples *t*-testing indicated that this difference did not reach statistical significance (*t*(42.8) = 1.76, *P* = .09), although the effect size was moderate (Cohen's d = 0.50). When comparing the 5 items with the highest item–total point-biserial correlations (top quintile) in each group, mean RPB was lower for AI-generated questions (0.34) than for human-generated questions (0.38). In contrast, the 5 lowest RPB AI-generated items demonstrated markedly poorer alignment with overall test performance, with a mean RPB of −0.06, compared with a mean RPB of 0.09 for the corresponding human-authored items.

**Table 2.** Item–Total Point-Biserial Correlation Coefficients (RPB) for AI-Generated and Human-Generated Pediatric Multiple-Choice Questions Administered to Participants.

	Human-Generated	AI-Generated
Number of items in exam	25	25
Number of examinees	159	159
Mean RPB (SD)	0.17 (0.18)	0.23 (0.15)
Items with negative RPB (%)	0	3 (12%)
Items with zero RPB (%)	0	0
Items with low RPB (%)	7 (28%)	14 (56%)
Total number of items with acceptable item–total correlation (%)	18 (72%)	11 (44%)

*Note.* Item–total point-biserial correlation coefficients (RPB) indicate the relationship between individual item performance and total test score. Positive values indicate appropriate item–test alignment, while negative values suggest poor item functioning. RPB indices ≥ 0.20 were considered acceptable.

### Item difficulty

The overall mean difficulty index for the examination was 0.62. AI-generated items ranged in difficulty from 0.07 to 0.93, with a mean ± SD difficulty index of 0.66 ± 0.21. Human-generated MCQs had range of difficulty from 0.19 to 0.95, with a lower mean ± SD of 0.59 ± 0.19 (Table 3). Independent-samples *t*-testing demonstrated no statistically significant difference in item difficulty between AI- and human-generated MCQs (*t*(47.7) = −1.21, *P* = .23), although the effect size suggested a small-to-moderate difference favoring more appropriate difficulty in human-authored items (Cohen's d = 0.34).

**Table 3.** Item Difficulty Indices for AI-Generated and Human-Generated Pediatric Multiple-Choice Questions Administered to Participants.

	Human-Generated	AI-Generated
Number of items in exam	25	25
Number of examinees	159	159
Mean score (%)	14.18 (57.6%)	16.42 (65.7%)
Mean difficulty % (SD)	0.56 (0.19)	0.66 (0.21)
Easy items (	7 (28%)	12 (48%)
Difficult items (%)	1 (4%)	2 (8%)

*Note.* Item difficulty index represents the proportion of students answering an item correctly. Difficulty values between 0.30 and 0.70 were considered acceptable.

### Reliability

Internal consistency reliability for the full 50-item examination yielded a Cronbach's alpha (KR-20) of 0.858. When analyzed separately, the AI-generated questions had acceptable but lower reliability (α = 0.758) than the human-generated item set (α = 0.765).

### Distractor efficiency

Both AI-generated and human-generated MCQs contained 100 distractors each. Of these, 47% of AI distractors were nonfunctioning (selected by fewer than 5% of examinees), compared with 35% of distractors in the human-generated group (Table 4). This difference was statistically significant (*P* = .04). At the item level, human-authored questions contained a greater mean number of functioning distractors (mean ± SD: 2.72 ± 1.18) compared with AI-generated items (2.12 ± 1.10). The difference reached statistical significance (*t*(45.8) = 2.02, *P* = .048), with a moderate effect size (Cohen's d = 0.51). Furthermore, over one-third of human-generated MCQs (36%) included 4 fully functioning distractors, compared with only 12% of AI-generated questions.

**Table 4.** Distractor Analysis for AI-Generated and Human-Generated Pediatric Multiple-Choice Questions Administered to Participants.

Criteria (%)	Human-Generated	AI-Generated
Number of items in exam	25	25
No of distractors assessed	100	100
Distractors with frequency = 0% *n* (%)	2 (2%)	9 (9%)
Distractors with frequency <5% *n* (%)	35 (35%)	47 (47%)
Functioning distractors per test *n* (%)	68 (68%)	53 (53%)
Functioning distractors per item *M* (*SD*):	2.72 (1.18)	2.12 (1.10)
Functioning distractors per item *n* (%):		
None	1 (4%)	1 (4%)
One	3 (12%)	8 (32%)
Two	7 (28%)	6 (24%)
Three	5 (20%)	7 (28%)
Four	9 (36%)	3 (12%)

*Note.* Distractor efficiency was evaluated by identifying nonfunctioning distractors, defined as options selected by fewer than 5% of examinees. Values are reported as the number and percentage of nonfunctioning distractors per group, mean number and the proportion of functioning distractors per item.

## Discussion

The aim of this study was to assess the capabilities of readily available generative AI software in the generation of quality MCQs for medical school exams and study resources. This is in the context of the increasing prevalence of ChatGPT in education generally, and for specific application in medical education.^33,34^

Assessment quality in medical education can be conceptualized through complementary theoretical lenses. Van der Vleuten's (1996) utility framework provides a practical extension by proposing that assessment quality is determined by the balance of five criteria—reliability, validity, educational impact, acceptability, and cost-effectiveness—rather than by any single dimension. Applying this framework allows for a nuanced interpretation of AI- versus human-generated MCQs, recognizing both their statistical performance and their broader implications for assessment design, educational value, and adoption in medical education.

From a reliability standpoint, both the AI-generated and human-generated question sets had “appropriate” (>0.7) reliability coefficients (0.758 and 0.765) with the combined set of questions having a “good” (>0.8) reliability measure of 0.858.^23^ This indicates that utilizing AI question generation allowed for the creation of an exam with appropriate test content, although it must be noted that there was a human element of the selection of AI questions for inclusion.

Overall, the AI-generated questions performed less consistently than human-authored items, with lower discrimination indices (mean 0.19 vs. 0.29) and a higher proportion of items falling outside acceptable ranges for both discrimination and difficulty. The AI also generated 3 defective items which had negative discrimination indices, while the worst discrimination human question had a discrimination index of 0.04. These findings raise concerns about the dependability of AI-generated questions for high-stakes assessment. In terms of validity, although some AI items fell within the ideal discrimination and difficulty parameters, the overall weaker performance suggests that AI currently lacks the capacity to consistently generate items that accurately measure the intended construct of pediatric medical knowledge.

Overall, the findings indicate that human-authored MCQs demonstrated more consistent psychometric quality than AI-generated items, reinforcing the continued importance of expert involvement in assessment design. Although both AI- and human-generated item sets achieved acceptable internal consistency, reliability alone is insufficient to establish assessment quality or validity.^35,36^

Differences in item discrimination were the most prominent and educationally meaningful. Human-authored items showed significantly higher discrimination indices, whereas a substantial proportion of AI-generated items failed to meet acceptable thresholds, including some with negative discrimination. These findings are consistent with emerging evidence that generative AI can produce plausible but psychometrically unstable assessment items, particularly without structured prompting or expert oversight.^14,24^

Item–total point-biserial correlations provided complementary validity evidence, with lower and occasionally negative RPB values observed among AI-generated MCQs, suggesting weaker alignment with overall test performance. In contrast, human-authored items demonstrated consistently positive item–test correlations, supporting stronger construct alignment. Differences in item difficulty did not reach statistical significance; however, AI-generated items tended to be easier and more variable, echoing findings from prior comparative studies of AI- and human-generated MCQs.^27,37^

Distractor analysis further differentiated the 2 item sets. AI-generated MCQs contained a higher proportion of nonfunctioning distractors and fewer items with fully functional distractor sets, a limitation likely contributing to reduced discrimination. Effective distractor construction remains a recognized challenge for automated item generation and is closely linked to assessment validity and educational impact.^11,32^

With regard to educational impact, the inferior distractor functioning in AI questions—more nonfunctioning distractors and fewer items with all distractors functioning—suggests reduced potential for challenging students’ higher-order cognitive reasoning. However, the ability of AI to generate some usable items highlights its promise as a supplementary tool that, with refinement, could enhance assessment banks and support student learning. The dimension of acceptability is more nuanced: while educators may remain cautious about fully adopting AI-generated items due to variability in quality, the technology may gain traction if integrated in hybrid workflows where humans provide oversight, content validation, and refinement. Finally, cost-effectiveness represents a potential advantage of AI, as its capacity to generate large volumes of items quickly could reduce faculty workload and resource demands, particularly if AI is used to suggest distractors or reframe stems for human-authored questions.^15,38^

Since AI remains a relatively new tool in medical assessment, considerable uncertainty still surrounds how it can most effectively assist human assessors in enhancing MCQ quality. Recent research suggests that generative AI can play a *supportive*, rather than purely substitutive role. For example, the “Hybrid Automatic Item Generation” approach, which combines AI-derived item templates with expert input, has been shown to speed up MCQ creation while preserving content validity; in one proof-of-concept, item-templates for pediatric MCQs were generated in only 10 min under expert oversight.^39^ Similarly, a recent tool for refining USMLE-style questions employed a universal prompt that allows learners to draft MCQs and then receive AI-assisted feedback on stems, distractors, alignment with learning objectives, and overall item structure. This tool was reported to improve learners’ question-writing and offer scalable improvement without heavy faculty burden.^40^ Another relevant study found that AI-generated questions tend to be easier on average and emphasize lower-order cognitive skills, but when paired with human review and editing, the AI questions could reach closer parity in discrimination indices.^24^ These findings underscore that while AI alone often underperforms humans in metrics like discrimination, difficulty, and distractor functioning, there is growing evidence for the viability of hybrid workflows.

Thus, hybrid human-AI workflows—where AI is used to generate draft stems, suggest plausible distractors, or rewrite poorly performing question stems, followed by human expert review—look particularly promising. Such workflows leverage the strengths of both: AI's speed, scalability, and ability to generate multiple alternatives; human experts’ domain knowledge, judgment concerning cognitive level, and capacity to ensure alignment with curricular aims. Integrating AI in this more guided, iterative way may help reduce the “noise” (nonfunctioning distractors, misaligned difficulty) found in many AI-only items, while preserving some of the efficiency gains. Minimal work has so far rigorously quantified how much hybrid workflows can improve discriminatory power, distractor performance, and educational impact compared to purely human-generated question sets.^38^ There is therefore a compelling case—supported by the early results of Kiyak et al and others—for designing such hybrid approaches in your own setting, to leverage the comparative strengths of both question generation methods.^24,39^

Taken together, these results suggest that while AI alone cannot yet replace human expertise in MCQ development, it holds promise as a complementary tool in hybrid human–AI workflows.^24,39^ This aligns with the utility framework's central premise: that no single dimension defines assessment quality, but rather that tradeoffs across reliability, validity, educational impact, acceptability, and cost-effectiveness must be balanced in context.

## Strengths and Limitations

The key strength of this study is the use of a cohort of medical students as test-takers, which allows the analysis of the generated questions within a realistic context which reflects the way the MCQs are used most in medical education. The use of a test taking cohort is also provided the ability to quantitatively compare the AI and human groups through item and distractor analysis. Additionally, a large sample size of 157 participants allows for sufficient power for the item analysis. Previous work assessing AI MCQ questions in a medical education context have assessed question quality using expert panels or student perception.^14,15^ Another strength of this study compared to previous work on generative AI MCQs is that respondents were not asked to rank the quality of the questions and were blinded as to the origin of the question.^14^ This addresses the issue that respondents may be inclined toward perceiving questions as higher or lower quality based on the belief that they are AI generated.^41^

The key limitation of this study involves the way the AI model was used to generate the MCQs. Since the AI was not provided with any reference material, only available online information was used to generate the questions which risk the use of unreliable sources. The use of AI tools to develop MCQs is a rapidly developing area with ongoing work being done to create methods to better leverage AI capabilities to generate questions and the development of AI platforms designed specifically to be used within an education setting.^38,42^ The methodology utilized in this study provides a way to compare different MCQ generation protocols to facilitate ongoing improvement of AI question generation. Using more specific prompts and training the AI model by providing source material has been shown to yield higher quality questions and future research should focus on assessing quality metrics for AI questions generated using more refined workflows. Selection of which AI-generated questions were included in the final exam was performed by a human to ensure a balanced examination with a range of topics, however, this also potentially impacted the quality of AI questions presented to the students. Additionally, a formal a-priori sample size calculation was not performed; however, the whole cohort participated in the study and the cohort size was sufficient for item-level psychometric analysis. Another limitation of this study was the online administration of the exam which made it challenging to ensure that external resources were not being used by students to assist in answering questions. This method was chosen as it allowed for an increased number of respondents with reduced resource requirements, however, it would be ideal to compare AI and human questions in a high stakes proctored exam setting to ensure results more accurately reflect formal examination performance.

## Conclusion

This study compared AI- and human-generated pediatric MCQs using CTT item analysis, revealing clear differences in quality. Human-authored items demonstrated stronger discrimination, more appropriate difficulty, and better distractor functioning, while AI-generated questions were less reliable overall, though a subset met acceptable standards. These findings suggest that generative AI, while efficient in producing large volumes of items, cannot yet replace human expertise in assessment design. Viewed through van der Vleuten's utility framework, AI offers advantages in cost-effectiveness but falls short in reliability, validity, and educational impact when used independently.
